# The use of electronic tools and the information highway to facilitate knowledge transfer in primary care

**DOI:** 10.1186/1710-1492-6-S4-A5

**Published:** 2010-12-10

**Authors:** Michel Labrecque

**Affiliations:** 1 Department of Family and Emergency Medicine, Université Laval, Québec, G1V 0A6, Canada

## 

Access to information is essential to effective and efficient clinical practice. On one hand, clinicians must keep track of new scientific evidence relevant to their field of practice. On the other hand, encounters with patients generate questions requiring answers in order to provide adequate clinical care. The main challenge is to access truly useful information that is: 1) relevant to clinical practice, 2) scientifically valid, and 3) requires minimal efforts to find, to appraise and to decide to integrate or not into practice. [1] The Internet now allows both staying up-to-date and finding answers to clinical questions.

Three main strategies can be used to take advantage of Internet resources: “push”, “pull” and “exchange.” The “push” strategy is similar to subscribing to a paper-based medical journal. The main purpose of this strategy is to keep aware of new knowledge relevant to practice. Many organizations offer to “push” clinical information to health care providers through Web portals (*e.g.,,* amc.ca, Journal Watch), e-mails alerts (*e.g.,,* InfoPratique, InfoPoem, evidenceupdates, Dynamed Weekly updates, GFD journal alerts, Pubmed) and RSS (Really Simple Syndication) (*e.g.,,* RSS4medics). There is, however, a risk of being rapidly overloaded with useless information, either irrelevant to practice, invalid, and/or needing extensive work to appraise, if pushed information is not judiciously selected.

Information can also be actively searched for (“pull” strategy), usually for the purpose of answering clinical questions. Most often, electronic tools offer a much faster and broader access to information than searching through piles of medical journals accumulating on shelves over the years. From databases of original studies (*e.g.,* Medline, Embase) and systematic reviews (*e.g.,* Cochrane Library) to virtual libraries (*e.g.,* MD Consult, Stat!Ref), repositories of clinical practice guidelines (*e.g.,* CMA Infobase, National Guideline Clearinghouse), critical summaries, synopsis of original articles (*e.g.,* InfoCritique, ACP Journal Club, InfoPoem, Clinical Evidence) and evidence-based e-textbooks (*e.g.,* UpToDate, Essential Evidence Plus, ACP PIER, Dynamed), a huge amount of clinical information can be accessed on the Web or on a personal digital assistant (PDA). Federated search engines searching simultaneous resources such as Infoclinique, TripDatabase and MacPlus can ease the process of finding answers to clinical questions in the maze of information. Most questions can be answered by a judicious combination of just a few of these resources.

Reaching out to colleagues to obtain or share information about a clinical question has always been valued by health care professionals. This “exchange” strategy for keeping up-to-date and answering clinical questions is increasingly available through Web 2.0 functionalities (blogs/tweeters, wikis, pod/podcasts). Website such as Asklepios from the Canadian Medical Association is an example of the multiple initiatives now available to foster communities of practice among health care providers who are sharing similar interests. However, we are still at the “early adapters” phase of implementation and the effectiveness of these new e-tools to improve healthcare has yet to be demonstrated. By combining selected “push” and “pull” resources, the internet can provide access to useful information to facilitate evidence-based clinical care.
